# The Story of Rose and Edward

**Published:** 1907-09

**Authors:** 


### THE STORY OF ROSE AND EDWARD.

Miss Rose—was a little over twenty-two. She was a bright, cheerful, happy girl, and this was her happiest day. Not only because on that day she was graduated from Banard with high honors, but Edward—dear Ed whom she loved and looked up to for so many years, had proposed last night, and the passion, romance and aroma of that months on their honeymoon, but they returned after about three proposal still lingered with her. And how the plans and hopes and dreams kept chasing each other in her fertile brain. She had decided where they should live, where they should spend their summer, how she should bring up her children, etc., etc. And Ed was a husband to be proud of. Tho' but twenty-eight years old he had already achieved eminence in the legal profession, and his practice was more than he could attend to. And he was one of those rare specimens, a truly honest lawyer. Not honest in the legal sense, but honest in the true human sense. And kind-hearted, a gentleman in the noblest sense of the word and an all-round athlete. A man to protect a woman from every possible care and make her happy as long as she lived. So thought Rose and she was right.

They were married in October. They expected to stay away three weeks. Rose was not feeling well, and traveling and staying in hotels didn't agree with her. She looked rather tired and fagged out, but that was natural. It was not natural, however, that after a week's rest she did not show any improvement. On the contrary, she began to look somewhat haggard. She had a little irritation in the genito-urinary tract, increased frequency of micturition, etc., but this is not unusual in newly married women, it was not considered necessary to consult a physician. Things continued this way, getting a little better and a little worse, until the beginning of January. On the fifth of January she was taken violently and dangerously ill. Severe abdominal pain, very rapid but hard pulse, and threatening collapse. The physician who was called in diagnosed the case as ruptured tubal pregnancy. A consulting surgeon was called in and it was decided that in order to save the patient's life, an immediate operation was necessary, and tho' it was midnight the patient was quickly removed to —'s private hospital and operated upon. No signs of extrauterine pegnancy were discovered, but about three and one-half pints of a blood-stained and somewhat purulent serum were removed. An examination of this serum demonstrated the presence of millions of gonococci. We have here to deal with a case of fulminant gonococcal salpingitis. Both tubes were thickened and inflamed and they were removed. And so was the now useless uterus. The operation was a "success," i. e., the patient recovered.

A confidential talk was had with Mr. Edward. He searched his tack of—he did not know whether it was gonorrhea or something memory for awhile—yes, some two years ago he had a very mild at- due to a "strain." It was very mild, it didn't bother him much, he went to his physician who gave him an injection and he was all right in three or four weeks. He never attached much importance to that attack and it had escaped his memory entirely. An examination of his urine, however, demonstrated the presence of shreds, and while no gonoccocci could be found in the urine, they were demonstrated in the expressed secretion from the prostate and seminal vesicles. The despair of Mr. Edward at learning that he was the unwitting cause of the tragedy can better be imagined than described.

Rose recovered, but you would hardly know her if you saw her. She aged ten years in ten weeks. She is making no plans, she has no hopes, she is dreaming no dreams—not for the present at any rate. Never again wil she be the happy Rose that he was before she became Mrs. Edward. Never will her home be gladdened by the noise, romp and laughter of little children.

Who is to blame? Nobody. Rose certainly is not, nor is Ed, for he certainly would have had his right hand cut off—and his left one, too—rather than cause the woman whom he loved above all else in the world any pain or suffering. But he "didn't know" and we cannot be blamed for things that we do not know, and that we never were told that we ought to know. Should we blame those who insist that all knowledge of sexual matters be kept away from the people? Perhaps, but even they are more to be pitied than blamed. For they are generally sincere in their beliefs and we cannot blame them for their ignorance.

No, nobody is to blame, but it is the duty of those who see the light to spread the knowledge of sexual matters and of the dangers of venereal disease before the people, so that tragedies like those htat have struck down our friends Rose and Edward may become rare or impossible in the future.

It would be an excellent plan if every man who indulged in promiscuous intercourse, no matter how rarely, had his urine and his prostatic secretion examined before marrying. This even if to his knowledge he never had gonorrhea. For there are gonorrheas without any subjctiv esymptoms, gonorrheas in which the gonococci remain dormant, only to awaken into virulent activity at the first opportunity. And newly married life is such an opportunity.—Critic and Guide, May, '07.

